# Isolation and Characterization of Two Lytic Phages Efficient Against Phytopathogenic Bacteria From *Pseudomonas* and *Xanthomonas* Genera

**DOI:** 10.3389/fmicb.2022.853593

**Published:** 2022-04-25

**Authors:** Nataliia Korniienko, Alla Kharina, Nikita Zrelovs, Barbora Jindřichová, Tomaš Moravec, Iryna Budzanivska, Lenka Burketová, Tetiana Kalachova

**Affiliations:** ^1^Institute of Experimental Botany of the Czech Academy of Sciences, Prague, Czechia; ^2^Educational and Scientific Center (ESC) “Institute of Biology and Medicine”, Taras Shevchenko National University of Kyiv, Kyiv, Ukraine; ^3^Latvian Biomedical Research and Study Centre, Rīga, Latvia

**Keywords:** bacteriophage (phage), *Pseudomonas syringae* pv. *tomato*, phytopathogenic bacteria, biocontrol, *Autographiviridae*

## Abstract

*Pseudomonas syringae* is a bacterial pathogen that causes yield losses in various economically important plant species. At the same time, *P. syringae* pv. *tomato* (*Pst*) is one of the best-studied bacterial phytopathogens and a popular model organism. In this study, we report on the isolation of two phages from the market-bought pepper fruit showing symptoms of bacterial speck. These *Pseudomonas* phages were named Eir4 and Eisa9 and characterized using traditional microbiological methods and whole-genome sequencing followed by various bioinformatics approaches. Both of the isolated phages were capable only of the lytic life cycle and were efficient against several pathovars from *Pseudomonas* and *Xanthomonas* genera. With the combination of transmission electron microscopy (TEM) virion morphology inspection and comparative genomics analyses, both of the phages were classified as members of the *Autographiviridae* family with different degrees of novelty within the known phage diversity. Eir4, but not Eisa9, phage application significantly decreased the propagation of *Pst* in the leaf tissues of *Arabidopsis thaliana* plants. The biological properties of Eir4 phage allow us to propose it as a potential biocontrol agent for use in the prevention of *Pst*-associated bacterioses and also as a model organism for the future research of mechanisms of phage–host interactions in different plant systems.

## Introduction

Phytopathogenic bacteria cause economically devastating diseases in a wide range of crops. To date, the most common strategies to treat plant bacterioses are the application of bactericides of different natures (from metal-containing organic compounds to plant-based extracts) (Bashan and de-Bashan, 2002; Li et al., 2017; Canzoniere et al., 2021; Elsharkawy et al., 2021), seed sterilization (Pyke et al., 1984) and coating with growth-promoting bacteria (Lugtenberg and Kamilova, 2009), or changing the fields to limit soil-borne contamination (Louws et al., 2001; Wilson et al., 2002). However, all these approaches have technical or economical limitations, and agriculture remains nonetheless in search of efficient and environmentally friendly alternative ways to protect plants from phytopathogenic bacteria. Bacteriophages (phages) are viruses of bacteria, highly specific to their hosts, self-reproducible, and self-eliminating. Phages have been proposed as biocontrol agents already shortly after their discovery by Twort (1915) and D’Herelle (1917). The high diversity of bacteriophages coupled with their host specificity gives a reason to optimistically think that any bacterial disease can be treated by phage application if only the right virus capable of infecting the respective etiologic agent of bacterial nature will be found (Svircev et al., 2018).

*Pseudomonas syringae* is a hemibiotrophic pathogen, infecting leaves or fruits through natural openings (stomata or accidental wounds) and propagating in the apoplastic space with the limited systemic distribution (Gullino et al., 2009; Xin and He, 2013). Being an epiphyte, *P. syringae* is transmitted from plant to plant under favorable conditions (high humidity, heavy rain, and mild temperature) and can survive on the leaf surface and in soil. Based on host specificity, the species has been subdivided into approximately 50 distinct pathovars (Gardan et al., 1999), and the list is currently extending as novel characterization methods emerge (Vieira et al., 2007). One of the most abundant pathovars, *P. syringae* pv. *tomato* (*Pst*) causes bacterial speck disease responsible for severe yield losses in tomatoes both in open fields and in greenhouses (Pernezny et al., 1995; Preston, 2000; Wilson et al., 2002; Lamichhane et al., 2015; Kolomiets et al., 2017). Given their importance, *Pseudomonas* spp. are among the most popular hosts for novel phage isolation (Zrelovs et al., 2021b). Moreover, novel phages infecting *P. syringae* are being constantly isolated and described, largely in search for appropriate *P. syringae* biocontrol agents among them (Korniienko et al., 2021). For example, a highly effective commercially available phage φ6 was found to be efficient against *P. syringae* pv. *syringae* in *in vitro* experiments (Pinheiro et al., 2019). This phage lately also appeared to be able to lyse *P. syringae* pv. *actinidae* (*Psa*) (Pinheiro et al., 2020), preventing the development of kiwifruit canker in both *in vitro* and *in vivo* setups. Another lytic *Psa* phage from the genus *Ghunavirus*, PPPL-1, was recently shown to be effective in preventing kiwifruit canker (Song et al., 2021). Five lytic phages of *P. syringae* pv. *porri* demonstrated their efficiency in the treatment of bacterial blight in leek (Rombouts et al., 2016). *P. syringae* pv. *morsprunorum* and *P. syringae* pv. *syringae* are known to cause cherry diseases. A subset of 13 specific lytic phages isolated from soil, bark, or leaf samples has shown promising effectiveness as a candidate phage cocktail against them (Rabiey et al., 2020).

Here, we report the isolation and characterization of two novel podophages, namely, *Pseudomonas* phages Eir4 and Eisa9, from pepper fruits with symptoms of bacterial speck. Phages were purified and accumulated on *Pst* DC3000, a rifampicin-resistant derivative of *Pst* DC52 (Cuppels, 1986). In this work, we have investigated the morphology, host range specificity, and biological properties of the newly isolated phages and their efficiency *in planta* and completely sequenced and annotated their genomes. Based on our observations, we believe that one of the phages described herein has a potential as biocontrol agent and can also be used as model phage for the research of mechanisms of phage action in triple interactions between phages, plants, and bacteria.

## Materials and Methods

### Bacteria Cultivation

*Pseudomonas syringae* pv. *tomato* DC3000 (*Pst*) were cultured at 28°C in Luria-Bertani (LB) medium (tryptone, 10 g/L; NaCl, 10 g/L; yeast extract, 5 g/L) containing 50 μg/ml rifampicin, in sealed tubes on a rotary shaker at 180 rpm or on Petri plates (in this case medium was supplemented with 1.4% agar). For growth rate evaluation, bacterial suspension was cultivated in 96-well plates. Growth was evaluated by continuously measuring the optical density of the culture at 600 nm wavelength (OD_600_) on TECAN Infinite^®^ 200 PRO (Switzerland).

### Phage Isolation From Natural Environment

Bacteriophages were isolated from pepper fruits with bacteriosis symptoms, typical for bacterial speck induced by *Pst* (Pernezny et al., 1995). The initial amount of bacteriophages in the samples was amplified by the enrichment method (Zaika et al., 2013; Kharina et al., 2015). The macerated tissue from brown rot lesions was transferred to liquid LB medium and incubated for 48 h at 28°C without shaking. After incubation, the suspension was centrifuged (3,400 *g*, 25 min, Thermo Heraeus Multifuge 3S Centrifuge, Sorvall 75006445 Swing Bucket Rotor), and the supernatant was mixed with chloroform 1:1 to remove bacteria. The upper aqueous phase was then plated on a bacterial lawn by the agar overlay method (Kropinski et al., 2009). Separate phage plaques were picked and transferred to 0.9% NaCl sterile solution (1 ml). Infectivity of isolated bacteriophages was tested on *Pst* by spot test. Briefly, *Pst* was incubated overnight in LB broth at 27°C and 180 rpm (OD_600_ = 3–4). Then, 200 μl of bacterial culture was added to 6 ml of 0.7% LB soft agar (preheated up to 45°C) and poured onto Petri dishes containing solid agar (LB medium supplemented with 1.4% agar) (both LB agar layers containing 50 μg/ml rifampicin). The plates were left to dry for 10 min. Subsequently, 5 μl of the phage lysate (10^9^ PFU/ml) was spotted on the bacterial lawn and incubated overnight at 28°C for formation of lysis zones.

### Phage Propagation and Purification

Phages were propagated and purified as described previously (Bonilla et al., 2016). Plaques of different morphology were picked from a plate with numerous negative colonies using a sterile Pasteur pipet and individually plaque-purified further. Plaques were resuspended into a microcentrifuge tube containing 1 ml SM buffer (5.8 g NaCl, 2.0 g MgSO_4_⋅7H_2_O, 50 ml 1 M Tris-HCl pH 7.4, in 1 L dH_2_O), vortexed for 5 min, and then centrifuged at 3,400 g for 5 min. Using a double-agar layer method, 10 confluent-lysis plates were obtained. Next, 5 ml of SM buffer were poured on top of the plates, and plates were gently shaken for 15 min at room temperature. Buffer from the top of the plates was collected and centrifuged at 4,000 *g* for 20 min. The resulting supernatant was filtered through a 0.22 μm syringe filter (Millex-GS Syringe Filter Unit, Millex, Germany), and 0.1 volumes of chloroform were added. Samples were vortexed and centrifuged again at 3,400 g for 5 min; the supernatant was then stored at 4°C. Phage titer was assessed by the agar overlay method. Phage samples were then concentrated by ultracentrifugation at 50,000 rpm for 6 h (The Optima™ XPN, Beckman Coulter, rotor 70-Ti). The pellet was resuspended in 1 ml of SM buffer.

### Negative Staining and Electron Microscopy of Bacteriophages

The samples were prepared for electron microscopy by applying 5 μl of concentrated purified virions (5.0 × 10^9^ PFU/ml) onto formvar-coated copper transmission electron microscopy (TEM) grids (3 mm, 300 mesh, Agar Scientific Ltd., Essex, United Kingdom) and incubated for 10 min at room temperature. Grids were then negatively stained with 2% uranyl acetate. Specimens were examined using the transmission electron microscope FEI (Morgagni 268D).

### Biological Properties of Phages

The phage host range was assessed by spotting 5 μl of phage suspension on plates with bacterial lawn, prepared similarly as for the phage purification and titer evaluation (Kropinski et al., 2009). The following bacterial strains were used: *Pseudomonas syringae* pv. *tomato* DC3000; *P. syringae* pv. *tomato* CUCPB5112 *hrcC*-, derived from *Pst* DC3000; *P. syringae* pv. *syringae* 4073; *P. syringae* pv. *morsprunorum* OPPB; *P. syringae* pv. *maculicola* 4326; *P. syringae* 8511; *P. syringae* pv. *syringae* 1022; *P. syringae* pv. *atrofaciens* 4394; *P. syringae* pv. *coronafaciens* 9030; *P. aeruginosa*; *P. putida* P-14; *P. putida* B-115T; *P. pseudoalcaligenes*; *P. fluorescens* DBM3506; *P. veronii* DBM3208; *P. fluorescens* cmm 2115; *P. chlororaphis* 4341; *Pantoea agglomerans* pv. *gypsophilae* 2406; *Agrobacterium rhizogenes* 3113; *A. tumefaciens* LBA4404; *Clavibacter michiganensis* ssp. *michiganensis* 7035; *Erwinia amylovora* 8/95; *E. amylovora* 5032; *E. carotovora* pv. *carotovora* IVIA 241/1; *Pectobacterium carotovorum*; *P. carotovorum* ssp. *carotovorum* 1077; *Ralstonia solanacearum* 2505; *Xanthomonas arboricola* pv. *juglandis* CCM 1449; and *X. vesicatoria* 2101 (Table 1). After 24 h incubation at 26°C, phage efficiency was evaluated by the presence/absence and relative size of the lysis zones on the bacterial lawn.

**TABLE 1:** Phage efficiency against epiphytic and rhizobiome-associated bacteria (spot test).

Bacterial strain	Eir4	Eisa9
*Pseudomonas syringae* pv. *tomato* DC3000	+ +	++
*Pseudomonas syringae* pv. *tomato* CUCPB5112 *hrcC-* (derived from *Pst* DC3000)	+ +	++
*Pseudomonas syringae* pv. *syringae* 4073	+ +	++
*Pseudomonas syringae* pv. *morsprunorum* OPPB	+ +	+
*Pseudomonas syringae* 8511	+	+
*Pseudomonas syringae* pv. *syringae* 1022	−	−
*Pseudomonas syringae* pv. *atrofaciens* 4394	+	+
*Pseudomonas syringae* pv. *coronafaciens* 9030	+	−
*Pseudomonas putida* P-14	+	–
*Pseudomonas putida* B-115T	+	−
*Pseudomonas aeruginosa*	−	−
*Pseudomonas pseudoalcaligenes*	−	−
*Pseudomonas syringae* pv. *maculicola* 4326	−	−
*Pseudomonas fluorescens* DBM3506	−	−
*Pseudomonas veronii* DBM3208	−	−
*Pseudomonas fluorescens* cmm 2115	−	−
*Pseudomonas chlororaphis* 4341	−	−
*Pantoea agglomerans* pv. *gypsophilae* 2406	−	−
*Agrobacterium rhizogenes* 3113	−	−
*Agrobacterium tumefaciens* LBA4404	−	−
*Clavibacter michiganensis* ssp. *michiganensis* 7035	−	−
*Erwinia amylovora* 8/95	−	−
*Erwinia amylovora* 5032	−	−
*Erwinia carotovora* pv. *carotovora* IVIA 241/1	+ +	−
*Pectobacterium carotovorum* ssp. *carotovorum* 1077	−	−
*Ralstonia solanacearum* 2505	−	−
*Xanthomonas arboricola* pv. *juglandis* CCM 1449	+ +	+
*Xanthomonas vesicatoria* 2101	+	+

*“++”: phage was efficient, lysis spots detected; “+” phage was efficient, small lysis spots detected; “−”: phage was not efficient.*

Phage adsorption dynamics was assessed as previously described (Adriaenssens et al., 2012). Briefly, overnight culture of *Pst* DC3000 in liquid LB medium supplied with 50 μg/ml of rifampicin was diluted to an OD_600_ = 0.4 and mixed with phage suspension at a multiplicity of infection (MOI) of 0.001. Immediately after mixing, 100 μl of the suspension was transferred into 2 ml tubes containing 850 μl LB medium with 50 μl chloroform. This stage was repeated every minute into the new tubes. Mixtures were then shaken for 10 min to remove bacterial debris. The supernatant was serially diluted (1:10), and the amount of non-adsorbed phages was assessed by the agar overlay method. Adsorption rate constant was calculated according to Rombouts et al. (2016) as [*k* = (2.3/(B × t)) × log(P0/P)], where B is the bacterial titer at time zero, P is the phage titer, and t is the time.

Phage thermal and pH stability was tested by incubating a phage suspension of 10^9^ PFU/ml in SM buffer at different temperatures (from -20 to 50°C) and in pH buffer ranging 1–13 (SM buffer adjusted to a given pH with NaOH or HCl) for 6 and 24 h and quantifying the phage titer by spot test.

Killing curves were obtained after infecting the *Pst* DC3000 (OD_600_ = 0.1) with phages at MOI of 0.1 and 0.01. Mixtures of *Pst* with the studied phages, as well bacterial and phage controls, were prepared in transparent 96-well plates and incubated at 26°C for 5 h on a microplate reader (TECAN Infinite 200 PRO, Switzerland); adsorption at λ = 600 nm was measured every 5 min.

The frequency of bacterial resistance was determined using the previously described method (Rombouts et al., 2016). Phage and bacteria were plated at MOI 10 to obtain the full lysis of the bacterial lawn on plates. After 72 h of incubation, emerging colonies were counted, and four independent colonies (two for each phage) were cultured and retested for resistance against both phages. The frequency of phage-resistant mutant occurrence was calculated by dividing the number of the resistant bacteria by the total number of sensitive bacteria.

### Phage Genomic DNA Extraction and Next-Generation Sequencing

Phage genomic DNA was extracted using the protocol for phage DNA extraction with phenol:chloroform (Center for Phage Technology, Texas A&M University). Briefly, phage lysates were precipitated with PEG (10% PEG-8000, 1 M NaCl final concentration) and incubated at 4°C overnight. The PEG-precipitated sample was centrifuged at 10,000 *g* for 30 min, and the pellet was resuspended in 5 mM MgSO_4_. The subsequent steps were the addition of 1.25 μl of DNase I and RNase (20 mg/ml) and incubation at 37°C for 1 h. Afterward, 1.25 μl of proteinase K of 20 mg/ml stock (20 μg total), 25 μl of 10% SDS stock (0.5% final concentration), and 20 μl of 0.5 M EDTA, pH 8.0 (20 mM final), were added, and the sample was incubated for 1 h at 60°C. Phenol:chloroform (1:1) was then added before centrifugation for several times, using decanted supernatants each successive time. Samples were incubated with 3 M NaOAc (pH 7.5) and 2.5 volumes of ice-cold ethanol (100%) overnight at -20°C. Next day, the samples were centrifuged at 10,000 *g* for 20 min in the bench centrifuge, and the pellet was washed with 70% ethanol twice. The pellet was then dissolved in TE buffer and stored in -20°C before proceeding with the next steps. The REPLI-g Single Cell Kit (Qiagen, Venlo, Netherlands) was used to increase the amount of DNA via whole-genome amplification (WGA) as per manufacturer’s instructions. To prepare the input for phage whole-genome sequencing, 200 ng of whole-genome-amplified phage genomic DNA was subjected to sonication using a Covaris S220 focused ultrasonicator (Covaris, Woburn, MA, United States) using a protocol for a target fragment length of 550 bp. Fragmented phage genomic DNA was then used to prepare DNA fragment libraries for 250 bp paired-end read sequencing using the TruSeq DNA Nano Low-Throughput Library Prep Kit (Illumina, San Diego, CA, United States) according to the manufacturer’s protocol. Adapters #2 and #4 from TruSeq DNA Single Indexes Set A (Illumina) were used for Eir4 and Eisa9 DNA fragment libraries, respectively. The resulting libraries were inspected for the quality and quantity of their contents using an Agilent 2100 bioanalyzer (Agilent, Santa Clara, CA, United States) with a High Sensitivity DNA Kit (Agilent), as well as a Qubit fluorometer (Invitrogen, Waltham, MA, United States) dsDNA high-sensitivity quantification assay (Invitrogen). Sequencing of the libraries was performed on the Illumina MiSeq system (Illumina) with a 500-cycle MiSeq Reagent Kit v2 nano (Illumina), with Eir4 and Eisa9 libraries being two of the 12 pooled libraries with different barcodes.

### Phage Genome *de novo* Assembly and Annotation

Demultiplexed raw read statistics of sequenced Eir4 and Eisa9 libraries were collected with the help of FastQC v0.11.9 (Andrews, 2010), revealing that the sequencing run has yielded 46,264 (Eir4) and 47,759 (Eisa9) read pairs. Read trailing base removal, as well as quality (bases below Phred quality of 20 removed) and length (50 bp minimum length of the read post-trimming) trimming, was performed using bbduk from the BBmap package (Bushnell, 2014). The trimmed read pairs were then subjected to *de novo* assemblies using Unicycler v0.4.8 in normal mode (Wick et al., 2017), yielding “circular” contigs of 40,177 and 40,730 bp length for Eir4 and Eisa9, respectively. Raw read mapping unto the assembled contigs representing complete genomes of the studied phages revealed that the mean depth of both is more than 550×.

These circular assemblies were then subjected to BLASTN analysis (Altschul et al., 1990) against all the complete viral genome sequences publicly available (nr/nt database restricted to virus taxid: 10239; other settings default). The total top-scoring hit to *Pseudomonas* phage JOR (MZ826346.1) with query coverage of 99% and 98.8% identity was documented for the Eir4 genome assembly as a query (*e*-value 0.0). The Eir4 genome assembly was then reverse-complemented and “linearized” to begin with base 30416 of the circular contig to ensure the assembled Eir4 genome collinearity with the complete genome of the already publicly available phage JOR. The circular assembly of Eisa9 was similarly reverse-complemented and “rearranged” to ensure the collinearity with the complete genome of the top-scoring *Pseudomonas* phage PollyC (NC_042104.1) hit, the alignment to which resulted in 87% query coverage of 77.34% identity when the Eisa9 complete genome was used as a query (*e*-value 0.0).

The rearranged linearized contigs representing complete phage genomes were then used as an input for an open reading frame (ORF) and tRNA gene prediction using tools available in the DNAmaster v5.23.6^1^ [for ORF prediction, Glimmer (Delcher et al., 2007) and GeneMark (Besemer and Borodovsky, 2005); for tRNA, Aragorn (Laslett and Canback, 2004) and tRNAscan (Lowe and Eddy, 1997)]. During the ORF prediction, four possible start codons (ATG, GTG, CTG, and TTG) were considered, and only ORFs encoding for a product with a length greater than 30 amino acids (aa) were documented and subjected to further supervised product functional annotation as described in Zrelovs et al. (2021a), with a modification that 3’-AUUCCUCCACUAG-5’ was used as an anti-Shine-Dalgarno sequence of *Pseudomonas syringae* pv. *tomato* (host species of phages Eir4 and Eisa9) for free_align.pl (Starmer et al., 2006).

### Packaging Strategy Prediction

For packaging strategy/expected genome physical termini type prediction, a set of TerL amino acid sequences from phages with an experimentally verified packaging strategy used previously by Merrill et al. (2016) was used to build a packaging strategy prediction maximum-likelihood tree based on TerL homology of different packaging-type employing phages with the addition of respective protein sequences from *Pseudomonas* phages Eir4 (UGL61101.1) and Eisa9 (UGL61147.1). Multiple amino acid sequence alignment of TerL proteins was performed using MAFFT v7.453 in automatic mode (Katoh and Standley, 2013), a maximum-likelihood phylogeny was reconstructed in IQ-TREE v 2.0.6. (Nguyen et al., 2015) under VT + F + R4 (Müller and Vingron, 2001; Soubrier et al., 2012) as the best-fit substitution model as determined by ModelFinder (Kalyaanamoorthy et al., 2017), while allowing for polytomies. Ultrafast bootstrap (UFBoot) with 1,000 replicates was used to determine the branch support (Minh et al., 2013). The resulting tree was midpoint rooted and visualized using FigTree v.1.4.4,^2^ and shapes of distal nodes of branches having less than 95% UFBoot support were manually removed using Inkscape v1.0.1.^3^

### Investigation of Evolutionary Relationships With Other Phages

To infer the genome nucleotide sequence evolutionary relationship of phages Eir4 and Eisa9 with other sequenced *Pseudomonas* sp.-infecting phages from the family *Autographiviridae* (podophages encoding their own RNA polymerase), the respective phage complete genome sequence accession numbers were retrieved from ICTV Virus Metadata Repository (VMR number 18, October 19, 2021, MSL36) for *Pseudomonas*-infecting phages belonging to the family *Autographiviridae* that are currently recognized by ICTV (hereafter referred to as VMR subset; *n* = 49). Accession numbers of the top 10 highest-scoring BLASTN hits not seen in this VMR subset were also retrieved for both Eir4 and Eisa9 complete genome queries. To be noted, for Eisa9, nine out of these top 10 non-VMR subset hits were phages of hosts other than *Pseudomonas* that had very little intergenomic similarity to Eisa9 but were nevertheless retained for context. After downloading the aforementioned phage complete genome entries, the resultant dataset consisting of 71 phage complete genome nucleotide sequences (*n* = Eir4 + 10 + Eisa9 + 10 + 49 = 71) was used as an input for VIRIDIC v.1.0 (Moraru et al., 2020) to calculate pairwise intergenomic distances between the phages.

Phylogenies were reconstructed for three of the selected phage Eir4 and Eisa9 marker proteins known to be evolutionary conserved among related *Autographiviridae* phages: major capsid protein (MCP; Eir4: UGL61088.1; Eisa9: UGL61137.1), terminase large subunit protein (TerL; Eir4: UGL61101.1; Eisa9: UGL61147.1), and RNA polymerase (RNAP; Eir4: UGL61062.1; Eisa9: UGL61131.1) using 22 presumed respective protein amino acid sequences (from phages Eir4 and Eisa9, as well as the 10 highest-scoring BLASTp hits against the respective Eir4 and Eisa9 protein amino acid sequence queries).

Multiple sequence alignments (MSAs) were performed using ClustalO v.1.2.4 in the automatical alignment option selection mode (Merrill et al., 2016), and the respective MCP, TerL, and RNAP MSAs were used as an input for maximum-likelihood phylogeny reconstruction in IQ-TREE v.2.0.6 (Nguyen et al., 2015) under WAG + G4 (Yang, 1994; Whelan and Goldman, 2001) (for MCP) and LG + G4 (Yang, 1994; Le and Gascuel, 2008) (for TerL and RNAP) as the best-fit amino acid substitution models according to the Bayesian information criterion after ModelFinder inference (Kalyaanamoorthy et al., 2017) and allowing for polytomies. Ultrafast bootstrap (UFBoot) with 1,000 replicates was used as a measure of branch support (Minh et al., 2013). Resulting trees were midpoint rooted and visualized using FigTree v.1.4.4 (see text footnote 2), and shapes of distal nodes of branches having less than 95% UFBoot support were manually removed using Inkscape v1.0.1 (see text footnote 3).

For pairwise genome nucleotide sequence/genome organization comparison of *Pseudomonas* phages Eir4 (OL581611) and Eisa9 (OL581612) and their closest known relatives with the standing in the current phage taxonomy (phage 17A (NC_048201) for Eir4 and PollyC (NC_042104) for Eisa9), as well as phage T7 (NC_001604; as arguably the most known and studied *Autographiviridae* phage), the respective complete annotated genomes were retrieved in the form of annotated GenBank entries. These entries were then manually color-coded by the addition of a “/color = R G B” qualifier for every ORF based on the functional annotation of its product as seen in the downloaded entries (without reannotating anything). Complete genomes of these selected phages (as well as Eir4 and Eisa9) with coding sequences colored based on their product function were then used as an input for Easyfig v.2.2.3 using the TBLASTX and BLASTN comparison options and drawing only the hits with a minimal length of 50 and maximum *e*-value of 1e-5 (Sullivan et al., 2011).

### Biological Activity *in planta*

Biological activity testing *in planta* was based on the approach of Álvarez et al. (2019). *A. thaliana* plants were cultivated on a solid medium and infected by flood inoculation (Ishiga et al., 2011). Stratified sterile seeds (*n* = 9–10 per plate) were cultivated on a solid MS/2 medium containing 2.2 g Murashige and Skoog medium (Duchefa, Netherlands). Seedlings were grown in Petri dishes for 12–14 days under a long-day photoperiod (16 h/8 h light/dark regime) at 100–130 μE m^–2^ s^–1^ and 22°C. On day 14, plates were flooded with a suspension of an overnight culture of *Pst* DC3000 in 10 mM MgCl_2_ (OD_600_ = 0.01) containing 6.25 μl of Silwet L-77 in 50 ml of suspension for 2–3 min and phage at MOI = 4. Control (mock) plants were flooded with phage suspension and buffer only. Samples were collected at 2 dpi (days post inoculation); each sample contained pooled tissues from rosettes of two to three plants (roots were cut off with scissors). Tissues were homogenized in 2 ml Eppendorf tubes with 1 g of 1.3 mm silica beads using a FastPrep-24 instrument (MP Biomedicals, United States). The homogenate was serially diluted in 10 mM MgCl_2_ and pipetted onto LB plates containing rifampicin. The colonies were counted after 1–2 days of incubation at 28°C.

## Results and Discussion

**FIGURE 1:**
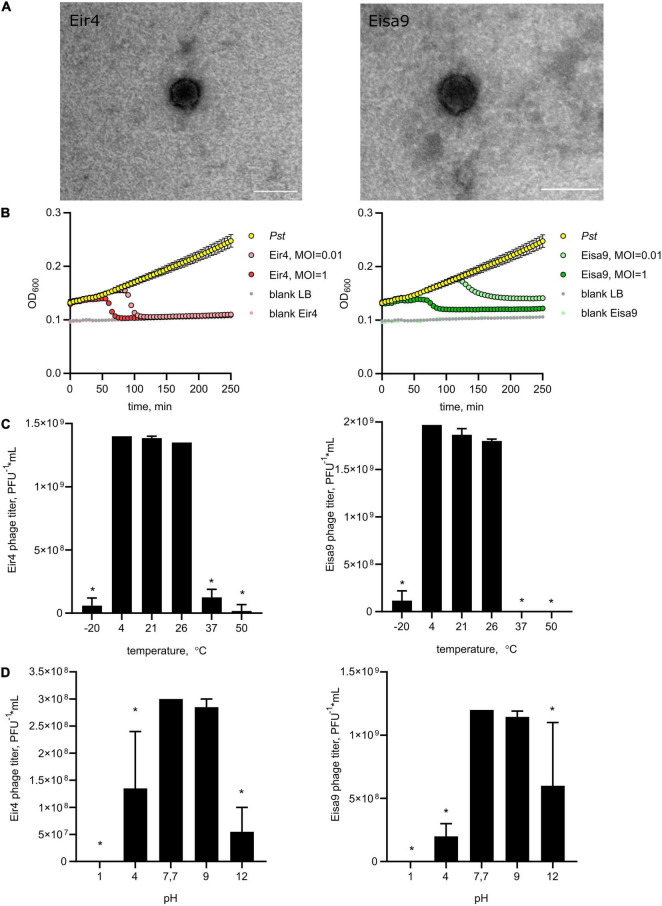
Morphology and biological properties of newly isolated phages of *P. syringae* pv. *tomato*, Eir4 (left panels) and Eisa9 (right panels). **(A)** Capsid morphology, transmission electron microscopy, scale bar = 100 nm. **(B)** Individual *in vitro* lysis kinetics for Eir4 and Eisa9 at an MOI of 0.01 or 1 on the *Pst* strain DC3000. Each value is the average from three different cultures ± standard deviation. **(C)** Temperature stability of phages. **(D)** pH stability of phages. Temperature stability tests were performed at pH = 7.7, and pH stability tests were performed at *t* = 4°C. Bar plots indicate the mean of two biological repeats + SEM; asterisks indicate statistically significant differences from values at *t* = 4°C **(C)** and pH = 7.7 **(D)**, Student’s *t*-test *p* < 0.05.

Two phages, named *Pseudomonas* phages Eir4 and Eisa9 were isolated from pepper plants with symptoms of bacterial speck. TEM revealed the icosahedral capsids of 55–60 nm with short tails (Figure 1A). After isolation and purification, the phages’ ability to lyse *Pst* DC3000 was tested. Both phages showed lytic activity against *Pst* DC3000, resulting in the formation of clear plaques on the bacterial lawn. Phage Eir4 formed bigger plaques (*d* = 4 mm) than did Eisa9 (*d* = 0.9–1 mm). Phages were amplified on *Pst* DC3000 culture. To estimate the potential of isolated phages in phage therapy, we tested the host specificity of Eir4 and Eisa9 against a range of plant pathogenic and growth-promoting bacteria. Both phages were proven efficient against *P. syringae* pv. *tomato* DC3000 and its mutant *Pst* DC3000 hrcC-. Interestingly, both Eir4 and Eisa9 were also efficient against other pathovars of *P. syringae*, like *syringae*, *atrofaciens*, and *morsprunorum*, but not *maculicola* (Table 1). Similarly, both phages formed lytic spots on the bacterial lawn of *Xanthomonas vesicatoria* (formerly named *P. vesicatoria*, or *P. gardneri*). Only Eir4 was found efficient against *P. syringae* pv. *coronafaciens*, *P. putida*, *X. arboricola*, and *Erwinia carotovora*. No lytic activity by any of the phages was detected on other tested Gram-negative (*P. fluorescens*, *P. veronii*, *P. chlororaphis*, *P. aeruginosa*, and *P. pseudoalcaligenes* and the more distant *E. amylovora*, *Ralstonia solanacearum*, *Pantoea agglomerans* pv. *gypsophiliae*, *Agrobacterium tumefaciens*, or *A. rhizogenes*) or Gram-positive bacteria (*Clavibacter michiganensis*). As both phages were isolated from the same biological material, it is not uncommon for them to have an overlapping host range. Similarly, as described by Martino et al. (2021), even rather distantly related phages isolated from the soil surrounding kiwifruit trees were able to lyse several strains of *P. syringae* pv. *actinidiae* and *phaseolicola*. Based on the abovementioned host range, both phages appear as efficient agents to potentially treat bacterioses caused by bacteria from *Pseudomonas* and *Xanthomonas* genera, with no impact on commensal and growth-promoting bacteria. This makes Eir4 and Eisa9 phages good candidates to be applied separately or as a part of phage cocktails for field use.

### General Characteristics of Eir4 and Eisa9 Phages

To investigate the dynamics of phage interaction with host bacteria, we performed adsorption tests and killing curve experiments. Both for Eir4 and Eisa9, 98% of phages were irreversibly adsorbed to the *Pst* host cell within 1 min, with adsorption rate *k*’ = 5.8 × 10^–10^ for Eir4 and 6.34 × 10^–10^ for Eisa9. This is quite similar to some other *Pseudomonas* phages (Sillankorva et al., 2008), but slower than phage φ6 (Vidaver et al., 1973). Killing curves were generated by monitoring the optical density of *Pst* suspension in liquid LB media at 600 nm (OD_600_) in the presence of phages at MOI = 1 and 0.01. In the presence of Eir4, OD_600_ rapidly decreased after 80 min (MOI = 1) and after 100 min (MOI = 0.01). For Eisa9, OD_600_ decreased after 125 min for MOI = 1 and after 150 min at MOI = 0.01 (Figure 1B). Interestingly, the OD_600_ decrease was only partial in the case of Eisa9, while the OD_600_ for Eir4 dropped to the values of blank samples. We hypothesize that it might be a result of a slower phage life cycle, so the high concentration of bacteria that propagated in media by the time of burst was sufficient to fill the culture with cell debris. Another possible explanation could be the surfacing of the slow-growing phage-resistant bacteria.

After incubation of full lysis plates for 72 h, we detected the emergence of phage-resistant bacterial colonies. For Eir4, an average of 116 ± 10 colonies was observed per plate, which corresponds to the frequency of resistance of 3.5 × 10^–6^, and for Eisa9, an average of 294 ± 5 resistant colonies appeared per plate, with the frequency of phage resistance emergence being 0.9 × 10^–5^. Four independent randomly selected phage-resistant cultures acquired from such colonies appeared to be cross-resistant to both Eir4 and Eisa9 phages, although the mechanism of such resistance remains yet to be elucidated.

Assessment of thermal stability revealed that both Eir4 and Eisa9 were stable at a range of moderate temperatures from 4°C (storage temperature) to 26°C (*Pst* growth). A marked decline in phage viability was observed after 24 h of preheating at 37°C or freezing at -20°C (Figure 1C). This corresponds to the optimal temperatures for virulence of *Pst* in greenhouse conditions on host tomato plants (Gullino et al., 2009). As for the pH stability, both phages maintained infectivity in neutral or slightly alkaline conditions (pH = 7–9), while lower or higher pH was significantly reducing phage titers after 24 h of incubation (Figure 1D). Both phages regularly produced clear plaques as an indication of a non-lysogenic cycle.

### Genome Analysis

Genomes of phages were sequenced, annotated, and deposited to GenBank as *Pseudomonas* phages Eir4 (accession OL581611) and Eisa9 (accession OL581612). Both genomes were similar in terms of genome length, GC% content that resembled that of their host (e.g., ∼58.4% GC content for *P. syringae* pv. *tomato* strain DC3000 chromosome complete sequence; NC_004578.1), coding capacities, and predicted ORF number (Table 2), as well as ORF functions (Supplementary Tables 1, 2). Although due to the WGA that was partaken before whole-genome sequencing to increase the amount of genomic phage DNA, the elucidation of the exact genome termini using raw read pile-up inspection onto the assemblies (e.g., using PhageTerm or manually) was impossible (bias of fragment non-randomness introduced by WGA); the completeness of the acquired phage genomes was implicated by the “circularity” of the genome *de novo* assemblies. Inside the virion, however, the genomes of studied phages Eir4 and Eisa9 should likely represent linear dsDNA molecules of 40,177 and 40,730 bp or longer (in case of short direct terminal repeat presence, which is the most likely packaging strategy employed by both Eir4 and Eisa9 based on the TerL aa sequence-based packaging strategy prediction; Supplementary Figure 2), so the beginning of both genomes was chosen according to the related phages already deposited in GenBank.

**TABLE 2:** Genome overview of the studied phages.

Phage	Accession	Genome length (bp)	GC content (%)	ORF count	Coding capacity (%)	ORF products with functional predictions	Closest sequenced phage relative			
							Phage	Genome length (bp)	Accession	Similarity*
Eir4	OL581611.1	40,177	57.28	52	94.76	31/52	JOR	40,531	MZ826346.1	97.49%
Eisa9	OL581612.1	40,730	59.24	51	95.80	25/51	PollyC	41,651	NC_042104.1	67.29%

Similarity (*) = highest total scoring BLASTN hit query coverage (%) × identity (%). BLASTN e-values for the hits to the closest sequenced phage relatives were equal to 0.

The presence of ORF coding for a DNA-dependent RNA polymerase in both of the genomes allowed us to classify both of these podophages as members of the phage family *Autographiviridae* (Adriaenssens et al., 2020). There is absence of almost any genomic nucleotide sequence similarity, as well as very little pairwise amino acid sequence identity, between the proteins with the same predicted function from Eir4 and Eisa9. This hinted at quite a distant evolutionary relationship between them, which is, given their enormous diversity, not that uncommon even for the phages infecting the same host and coming from the same source as in the case of Eir4 and Eisa9.

**FIGURE 2:**
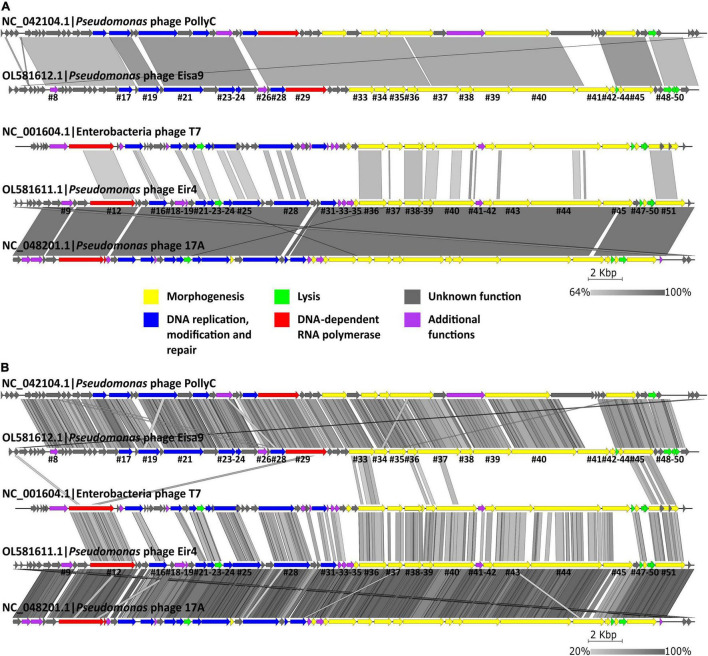
Pairwise genome nucleotide sequence comparison of *Pseudomonas* phages Eir4 and Eisa9, their respective closest-known relatives with standing in the current phage taxonomy, as well as an exemplar *Autographiviridae* phage T7 using BLASTN **(A)** and TBLASTX **(B)**. Genome representations are drawn to scale with the scale bar indicating 2,000 bp. Arrows represent ORFs and point in the direction of transcription. Color coding is based on the functional groups of the respective ORF putative products according to the legend, and ORFs of Eir4 and Eisa9 with functional prediction are annotated by the order of their appearance in the genome (Supplementary Tables 1, 2). Gray boxes represent regions of similarity between the genomes and are colored in gradient, with darker shades of gray representing higher region identity. The figure was generated using Easyfig (Sullivan et al., 2011).

The genome organizations of Eir4 and Eisa9 differed too. Eir4 genome organization was reminiscent of that from arguably the most well-known *Autographiviridae* phage—T7—with the ORF being responsible for encoding RNAP preceding a presumable DNA replication, modification, and repair functional ORF module, which also contains a lysozyme-encoding ORF in between other non-functionally related protein-encoding ORFs and is followed by ORFs encoding proteins necessary for virion morphogenesis, with the remainder of lysis proteins being located within it at the 3’ end of the genome, with a terminase small subunit located between the holin- and spanin-encoding ORFs and the TerL-encoding ORF following the spanin-encoding ORFs as the last one in the morphogenesis functional module and the penultimate ORF in the genome of Eir4 (Figure 2 and Supplementary Table 1). The genome of phage Eisa9, however, has an organization that is quite different—the RNAP-encoding ORF is preceded by the presumable DNA replication, modification, and repair functional ORF module that does not contain any lysis-associated genes within it and is followed by a morphogenesis ORF module which contains an ORF encoding holin before two of the module’s last ORFs (which encode both terminase subunits), with endolysin and both i- and o-spanin-encoding ORFs following the morphogenesis module after two hypothetical protein-encoding ORFs (Figure 2 and Supplementary Table 2). Neither of the phages was found to encode any proteins necessary for temperate life cycle, complementing the microbiological observations that these phages are strictly lytic. No known AMR genes or putative virulence factors beneficial to the host were found to be encoded by either of the phages as well. The genome contents of Eir4 and Eisa9 suggest that both of these phages look like suitable candidates to be further evaluated for possibilities of their application in *P. syringae* pv. *tomato* phage-mediated biocontrol.

**FIGURE 3:**
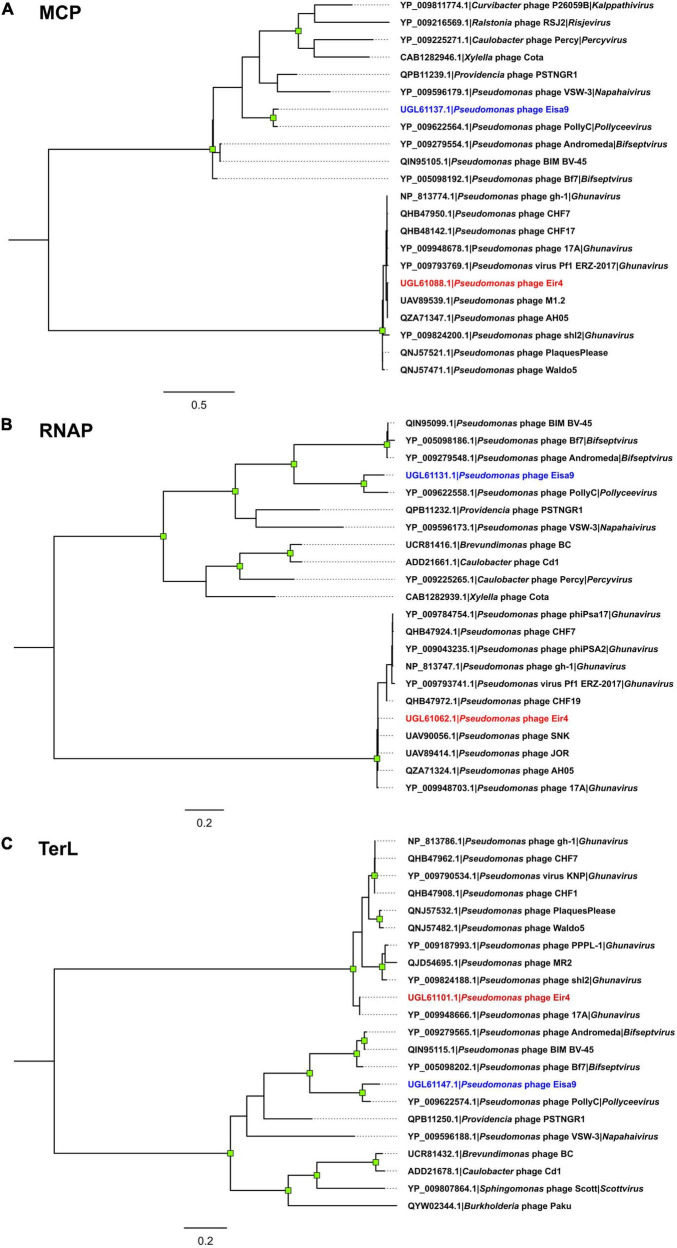
Maximum-likelihood trees of the selected *Pseudomonas* phage Eir4 and Eisa9 protein amino acid sequences and the 10 most related sequences found in the proteomes of other completely sequenced phages: **(A)** major capsid protein; **(B)** DNA-dependent RNA polymerase; **(C)** terminase large subunit. All the trees are drawn to scale, and branch lengths correspond to the number of amino acid differences per site. Tips are labeled as “protein accession | originating phage | genus phage belongs to (if it is currently recognized by the official phage taxonomy),” labels of the tips representing the amino acid sequences of Eir4 and Eisa9 proteins are colored in blue and red, respectively. The trees are midpoint rooted, and the distal nodes of branches with ≥ 95% ultrafast bootstrap support (out of 1,000 replicates) are indicated by green squares.

Investigation of possibilities for lesser taxon assignments for phages described herein revealed that Eisa9’s closest-known phage relative is the *Pseudomonas* phage PollyC, which is so far the only representative of *Pollyceevirus* virus genus. The intergenomic similarities between Eisa9 and PollyC (67–68%; Supplementary Figure 1; BLASTN *e*-value 0.0), however, fall behind the currently accepted genomic nucleotide sequence similarity genus-level assignment criterion (≥ 70%) for phages (Turner et al., 2021). In contrast, based on the phylogenies of the selected marker genes (Figure 3), as well as more-detailed genome organization and sequence comparison (Figure 2), Eisa9 might still be somewhat of an “edge case,” meaning that Eisa9 could potentially be proposed as an exemplar isolate of a novel species within the existing *Pollyceevirus* genus, rather than being viewed as a sole representative of a putative new phage genus. However, further analyses regarding the Eisa9 taxonomical standing are more fit for an ICTV taxonomic proposal and are out of the scope of this paper.

As for phage Eir4, based on the performed analyses, many closely related phages were found. Eir4 thus can convincingly be considered a member of the *Studiervirinae* subfamily and *Ghunavirus* genus, with its closest known relative having a standing in the current phage taxonomy being *Pseudomonas* phage 17A (∼91% intergenomic similarity with Eir4; BLASTN *e*-value 0.0). Yet, surprisingly, we found at least eight more phages without current standing in the official phage taxonomy available at public biological sequence repositories that show striking similarity to Eir4 (≥ 95% intergenomic similarity—isolates of the same species; Supplementary Figure 1; Turner et al., 2021).

Interestingly, the metadata associated with their annotated complete genome submissions indicate a very broad geographical distribution of such phage isolates that were previously recovered mostly from water sources in contrast to plant material (as in the case of phage AH05 and Eir4 within this study), which can definitely be proposed as different isolates belonging to the same novel phage species (Supplementary Figures 1, 3 and Table 3). Moreover, Roary pangenome analysis (Page et al., 2015), performed on the annotated genomes of the isolates seen in Table 3 under default settings (minimum percentage identity for BLASTp being 95%), has shown that the pangenome of representatives of this novel phage species encodes for 64 proteins with 44 of them being present in all nine of the isolates (including Eir4), while 54 of the products are present in two or more genomes. Speaking of Eir4 genome contents in this regard, only products of ORFs #1 (hypothetical protein), #45 (predicted tail fiber protein), #46 (hypothetical protein), and #50 (predicted o-spanin) from Eir4 were considered singletons under these rather conservative pangenome analysis settings. Of note, we did not manage to find any annotation for an o-spanin in the genomes of other phages presented in Table 3 (Supplementary Figure 3), which seems to be simply overlooked by the annotators of these phages, as it is an ORF enclosed within i-spanin-encoding ORF in the genome of Eir4 (as well as well-known phage T7), and predicting spanins usually requires particular attention from an annotator.

**TABLE 3:** Metadata associated with the *Pseudomonas* phage isolate Eir4 described in this study and other very closely related *Pseudomonas* phage isolates showing intergenomic nucleotide similarities above the species level demarcation (≥ 95%).

Accession	Phage	Genome length (bp)	Total BLASTN score	Similarity*	Isolation source	Country	Collection date	Host
OL581611.1	*Pseudomonas* phage Eir4	40,177	72,809	N/A (100%)	Pepper fruit	Ukraine	2013-08	*Pseudomonas syringae* pv. *tomato* str. DC3000
MZ826346.1	*Pseudomonas* phage JOR	40,531	69,286	97.49%	Jordan river	United States: Utah	2008-08-11	*Pseudomonas syringae* pv. *tomato* 99TK
MZ826354.1	*Pseudomonas* phage SNK	40,818	67,499	96.57%	River water	United States: Wyoming	2008-07-28	*Pseudomonas syringae* pv. *tomato* 99TK
MZ826351.1	*Pseudomonas* phage NOI	40,496	68,163	96.00%	Wastewater treatment plant	United States: Arizona	2007-03-13	*Pseudomonas syringae* pv. *tomato* 99TK
MZ826349.1	*Pseudomonas* phage M3.1	40,502	68,161	95.93%	Water source	United States	Not provided	*Pseudomonas syringae* pv. *tomato* 99TK
MZ826348.1	*Pseudomonas* phage M1.2	40,502	68,157	95.93%	Water source	United States	Not provided	*Pseudomonas syringae* pv. *tomato* 99TK
MZ826347.1	*Pseudomonas* phage M1.1	40,502	68,157	95.93%	Water source	United States	Not provided	*Pseudomonas syringae* pv. *tomato* 99TK
MZ501272.1	*Pseudomonas* phage AH05	40,502	68,157	95.93%	Leaf of horse chestnut tree	United Kingdom	2011-09-01	*Pseudomonas syringae*
MZ826344.1	*Pseudomonas* phage ALEA	40,502	68,148	95.93%	Raw influent sewage	United States: Minnesota	2006-11-02	*Pseudomonas syringae* pv. *tomato* 519

Similarity (*) = respective BLASTN hit query coverage (%) × identity (%) using Eir4 as query. BLASTN expect values for the hits were equal to 0. Collection dates are given in the “yyyy-mm-dd” date format.

### Efficiency of Eir4 and Eisa9 *in planta*

**FIGURE 4:**
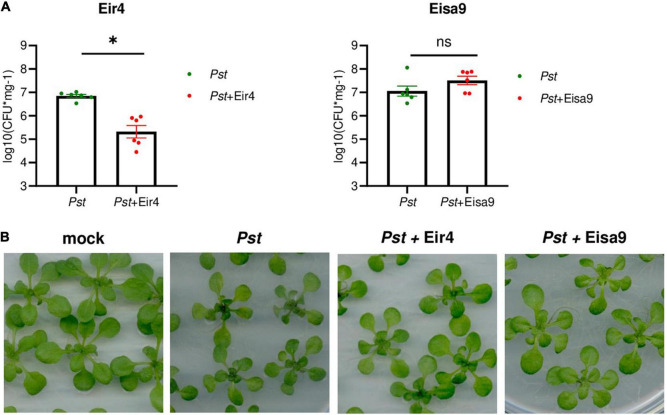
Activity of Eir4 and Eisa9 against *Pst in planta*. **(A)** Efficiency of Eir4 (left panel) and Eisa9 (right panel) in a flood inoculation setup. Two-week-old seedlings of *Arabidopsis thaliana* Col-0 were flood-inoculated with a mixture of a phage with *Pst*. At 2 dpi, leaf tissues were harvested for quantification of bacteria propagated within tissues. Bars represent the mean ± SEM, and circles represent individual values. An asterisk indicates statistically different variants, *p* < 0.05, Student’s *t*-test, *n* = 6. **(B)** Representative images of *A. thaliana* plants treated with *Pst* or a mixture of Pst + Eir4 and Pst + Eisa9, flood inoculation setup, 2 dpi (bar 10 mm). Mock: 10 mM MgCl_2_.

In order to investigate the efficiency of phages *in planta*, we took advantage of the well-studied interactions between *Pst* and *Arabidopsis thaliana* plants. Two-week-old *A. thaliana* Col-0 plants were flood-inoculated with the *Pst* or a mixture of *Pst* and phages to model the potential field application of phage preparations. In this setup, phages are premixed with bacteria immediately before the inoculation, so part of the phage is potentially being adsorbed on bacteria before contacting the plant. However, the relatively low MOI requires phage propagation *in planta* to successfully inactivate all bacteria. At 2 dpi, whole plant rosettes were harvested for the quantification of bacteria that propagated inside leaf tissues (Figure 4A). In samples inoculated with a mixture of *Pst* with phage Eir4, the load of *Pst* was significantly lower in comparison to plants treated with *Pst* only. The presence of phage Eisa9, however, did not influence *Pst* propagation *in planta*. Having no significant decrease in *Pst* growth in the presence of Eisa9 might be a result of a slower phage life cycle (burst occurring only after 120 min at MOI = 1, Figure 1B) or the contribution of newly selected resistant bacteria (as the frequency of resistance emergence appeared to be more than twofold higher for Eisa9). However, while *Pst*-inoculated plants exhibited growth reduction and leaf yellowing in comparison to mock-treated ones, both *Pst* + Eir4 and *Pst* + Eisa9 plants showed less yellowing and almost normal growth (Figure 4B). In the frame of potential field application, this suggests that both Eir4 and Eisa9 are good candidates for future field trials as they repress the development of *Pst*-caused disease symptoms, even though only Eir4 significantly reduced the bacterial propagation within the plant tissues. The mechanism of the Eisa9 effect on disease development, thus, remains to be investigated further.

## Conclusion

In the present study, *Pseudomonas* bacteriophages Eir4 and Eisa9 were first identified and characterized. Both phages had the ability to cause lysis of different isolates of *P. syringae* pv. *tomato* and several other closely related strains belonging to *Pseudomonas* and *Xanthomonas* genera. Additionally, Eir4 was efficient against *Erwinia carotovora* pv. *carotovora*. Biological characterization and genomic analysis showed that these phages had significant similarities with the previously reported phages against bacteria of *Pseudomonas* genus. General characteristics, i.e., adsorption constant and physical stability, were also similar to previously reported *Pst* phages. Genome analysis revealed that Eir4 and Eisa9 were rather similar in terms of genome length, GC% content, coding capacities, and predicted ORF number, as well as ORF functions. The presence of ORF coding for a DNA-dependent RNA polymerase in both of the genomes allowed us to classify both of these podophages as members of the phage family *Autographiviridae*. Eisa9’s closest-known phage relative with official standing in the current phage taxonomy appeared to be the *Pseudomonas* phage PollyC (intergenomic similarities between Eisa9 and PollyC are about 67–68%), which was so far a single phage representing the *Pollyceevirus* genus. As for Eir4, the closest-known currently taxonomically recognized relative was *Pseudomonas* phage 17A (∼91% intergenomic similarity) from the *Ghunavirus* genus. However, sequences of a notable amount of yet taxonomically unrecognized and geographically and ecologically diverse *Pseudomonas syringae*-infecting phage isolates boasting a phage species level similarity to Eir4 can be found in public biological sequence repositories, hinting at the success of Eir4-like phages in a wide array of natural environments. As for the practical application, only Eir4, but not Eisa9, significantly reduced the propagation of bacteria *in planta*. Considering these results, Eir4 could be recommended for phage treatment of *Pst*-induced infections in plants, but also as a model object for deeper investigation of interactions between phages and bacteria inside the plant host.

## Data Availability Statement

The datasets presented in this study can be found in online repositories. The names of the repository/repositories and accession number(s) can be found in the article/Supplementary Material.

## Author Contributions

NK, TK, and AK designed the study. NK, AK, and IB isolated and purified bacteriophages. TM performed the electron microscopy. NK isolated the phage DNA. NZ performed the phage genome sequencing and bioinformatic analyses. NK, TK, BJ, and LB performed the biological characterization of the phages. NK, NZ, and TK wrote the manuscript and prepared the figures and supplementary materials. All authors commented and approved the manuscript.

## Conflict of Interest

The authors declare that the research was conducted in the absence of any commercial or financial relationships that could be construed as a potential conflict of interest.

## Publisher’s Note

All claims expressed in this article are solely those of the authors and do not necessarily represent those of their affiliated organizations, or those of the publisher, the editors and the reviewers. Any product that may be evaluated in this article, or claim that may be made by its manufacturer, is not guaranteed or endorsed by the publisher.

## Footnotes

1
https://phagesdb.org/DNAMaster/

2
http://tree.bio.ed.ac.uk/software/figtree/

3
https://inkscape.org

## Supplementary Material

Supplementary Figure 1 Viridic heatmap showing pairwise intergenomic distances (%) of *Pseudomonas* phages Eir4 and Eisa9 with other related phages. Labels of *Pseudomonas* infecting *Autographivirdae* phages with a standing in the official phage taxonomy (subset from the ICTV virus metadata resource; n = 49) are in black font color. Labels of ten phages most similar to Eir4 that were not found as a part of *Pseudomonas*-infecting *Autographiviridae* phage VMR subset are in red font color (n = 10), label of Eir4 is in red and is also made bold. Labels of ten phages most similar to Eisa9 that were not found as a part of *Pseudomonas*-infecting *Autographiviridae* phage VMR subset are in blue font color (n = 10), label of Eisa9 is also made bold. Red rectangle indicates a cluster of phage genomes that are either officially recognized as representatives of genus *Ghunavirus* or can be proposed for inclusion in it. Maroon rectangle indicates *Pseudomonas* phage isolate genomes that belong to the yet unproposed same phage species as Eir4. Purple rectangle indicates *Pseudomonas* phages PollyC and Eisa9, which can probably be proposed as an exemplar isolate for creation of a novel species in the *Polyceevirus* genus.

Supplementary Figure 2 Terminase large subunit protein amino acid sequence maximum-likelihood tree for prediction of packaging strategy employed by phages Eir4 and Eisa9. The tree is drawn to scale, branch lengths correspond to the number of amino acid differences per site. Tips are labeled as “protein accession |originating phage |packaging strategy/genome termini type employed by the phage”, labels of the tips representing the amino acid sequences of Eir4 and Eisa9 proteins are coloured in red and blue, respectively. The tree is midpoint rooted and the distal nodes of branches with >=95% UFBoot support (out of 1000 replicates) are indicated by green squares. Maroon rectangle indicates the clade containing MRCA of T7 type SDTR packaging strategy employing phages and its children. “Unknown*” indicates that the TerL sequence comes from a phage for which the packaging strategy is unknown. LDTR stands for long direct terminal repeats, SDTR – for short direct terminal repeats.

Supplementary Figure 3 Pairwise genome nucleotide sequence comparison of *Autographivirdae* phage T7 as an exemplar *Autographivirdae* phage, phage 17A (representative of *Ghunavirus* genus) as the closest Eir4 relative with the standing in the current phage taxonomy, *Pseudomonas* phage Eir4 itself and eight phages that belong to the same putative novel species in genus *Ghunavirus* as Eir4 using BLASTN. Genome representations are drawn to scale with the scale bar indicating 2000 base pairs. Arrows represent open reading frames and point in the direction of transcription. Color-coding is based on the functional groups of the respective ORF putative products according to the legend and ORFs of Eir4 with functional prediction are annotated by the order of their appearance in the genome (Supplementary Table 1). Gray boxes represent regions of similarity between the genomes and are coloured in gradient with darker shades of gray representing higher region identity. The figure was generated using EasyFig (Sullivan et al., 2011).

Supplementary Table 1 Predicted ORFs and their functional annotation in the genome of *Pseudomonas* phage Eir4. Putative Shine-Dalgarno sequences 20 bases upstream of the predicted start codon of each ORF that are complementary to the 16S rRNA tail of *Pseudomonas syringae* pv. tomato (host) are underlined and highlighted in bold. Color coding for ORF product functional groups is as follows: yellow – proteins involved in virion morphogenesis; red—DNA-dependent RNA polymerase; blue—DNA replication, modification and repair; green—lysis; purple—additional functions; gray—hypothetical proteins of unknown function.

Supplementary Table 2 Predicted ORFs and their functional annotation in the genome of *Pseudomonas* phage Eisa9. Putative Shine-Dalgarno sequences 20 bases upstream of the predicted start codon of each ORF that are complementary to the 16S rRNA tail of *Pseudomonas syringae* pv. tomato (host) are underlined and highlighted in bold. Color coding for ORF product functional groups is as follows: yellow – proteins involved in virion morphogenesis; red—DNA-dependent RNA polymerase; blue—DNA replication, modification and repair; green—lysis; purple—additional functions; gray—hypothetical proteins of unknown function.
