# Supplementary material for: Isolation and Characterization of Two Lytic Phages Efficient Against Phytopathogenic Bacteria From Pseudomonas and Xanthomonas Genera
Source: Front Microbiol. 2022 Apr 25;13:853593. doi: 10.3389/fmicb.2022.853593 (PMC9083414; doi:10.3389/fmicb.2022.853593)
Supplement: Supplementary Figure 1 — Viridic heatmap showing pairwise intergenomic distances (%) of Pseudomonas phages Eir4 and Eisa9 with other related phages. Labels of Pseudomonas infecting Autographivirdae phages with a standing in the official phage taxonomy (subset from the ICTV virus metadata resource; n = 49) are in black font color. Labels of ten phages most similar to Eir4 that were not found as a part of Pseudomonas-infecting Autographiviridae phage VMR subset are in red font color (n = 10), label of Eir4 is in red and is also made bold. Labels of ten phages most similar to Eisa9 that were not found as a part of Pseudomonas-infecting Autographiviridae phage VMR subset are in blue font color (n = 10), label of Eisa9 is also made bold. Red rectangle indicates a cluster of phage genomes that are either officially recognized as representatives of genus Ghunavirus or can be proposed for inclusion in it. Maroon rectangle indicates Pseudomonas phage isolate genomes that belong to the yet unproposed same phage species as Eir4. Purple rectangle indicates Pseudomonas phages PollyC and Eisa9, which can probably be proposed as an exemplar isolate for creation of a novel species in the Polyceevirus genus. [file Data_Sheet_1.pdf]

Supplementary Material

Supplementary table 1. Predicted ORFs and their functional annotation in the genome of *Pseudomonas* phage Eir4. Putative Shine-Dalgarno sequences 20 bases upstream of the predicted start codon of each ORF that are complementary to the 16S rRNA tail of *Pseudomonas syringae* pv. tomato (host) are underlined and highlighted in bold. Color coding for ORF product functional groups is as follows: yellow - proteins involved in virion morphogenesis; red—DNA-dependent RNA polymerase; blue —DNA replication, modification and repair; green — lysis; purple — additional functions; gray — hypothetical proteins of unknown function.

ORF	Strand	Start position (bp)	End position (bp)	Product size (aa)	Shine-Dalgarno sequence		Start codon	Protein accession	Predicted function
					20 bp upstream of start codon (G-U wobble base pairs allowed)	DeltaG (kcal/mol; as calculated by free_align.pl -o "20bp" AUUCCUCCACUAG)			
#1	+	117	278	53	GGTTCCTTC**<u>GAGGG</u>**CATCTC	-4,046925	ATG	UGL61051.1	hypothetical protein
#2	+	434	559	41	A**<u>TGGTT</u>**GACAATCCTCTGAG	-2,18726	ATG	UGL61052.1	hypothetical protein
#3	+	809	934	41	GGCAACCAT**<u>TGAGGA</u>**CTCAC	-5,87344	TTG	UGL61053.1	hypothetical protein
#4	+	931	1185	84	TCACTG**<u>AAGGA</u>**TGCAACATC	-4,53508	ATG	UGL61054.1	hypothetical protein
#5	+	1185	1382	65	GCTGAC**<u>AAGGAG</u>**CCTGAGTG	-6,610015	ATG	UGL61055.1	hypothetical protein
#6	+	1387	1698	103	TCACCAG**<u>AAGGG</u>**CTAACATC	-3,71267	ATG	UGL61056.1	hypothetical protein
#7	+	1788	2267	159	CAACAAACGA**<u>AGGT</u>**AGCACC	-3,49838	ATG	UGL61057.1	hypothetical protein
#8	+	2298	2840	180	CGCTGTGAT**<u>AGGTG</u>**TTTTCA	-5,595345	ATG	UGL61058.1	hypothetical protein
#9	+	2837	3541	234	ACCTTTC**<u>AGGGG</u>**ATGACAGC	-4,90869	ATG	UGL61059.1	putative hydroxylase
#10	+	3538	3801	87	GTGCATT**<u>GGAG</u>**TGAAATCAA	-3,60793	GTG	UGL61060.1	hypothetical protein
#11	+	3804	4403	199	TACC**<u>GGGGAGG</u>**CTTCTAACC	-9,478722	ATG	UGL61061.1	hypothetical protein
#12	+	4543	7200	885	AACCCATTC**<u>GAG</u>**ACGACTTA	-0,359835	ATG	UGL61062.1	DNA-directed RNA polymerase
#13	+	7214	7351	45	TGCA**<u>TAAGGA</u>**AACCAATCAA	-5,867005	ATG	UGL61063.1	hypothetical protein
#14	+	7348	7617	89	CGTC**<u>AGGAG</u>**TGGAACGAGGA	-5,682865	ATG	UGL61064.1	hypothetical protein
#15	+	7617	8000	127	TTCCTG**<u>AAGGAG</u>**ACCAATTA	-6,610015	ATG	UGL61065.1	hypothetical protein
#16	+	8012	9079	355	GCCAAG**<u>TAAGGAG</u>**CAACCCA	-7,94194	ATG	UGL61066.1	ATP-dependent DNA ligase
#17	+	9273	9530	85	CCCTCA**<u>AAGGAG</u>**ACTCAACG	-6,610015	ATG	UGL61067.1	hypothetical protein
#18	+	9527	10171	214	ATCGTG**<u>AAGGAGGTG</u>**TTCGC	-14,20565	ATG	UGL61068.1	kinase

#19	+	10168	10338	56	CGCAACC**AAGGAG**TAATCGA	-6,610015	ATG	UGL61069.1	bacterial RNA polymerase inhibitor
#20	+	10335	10697	120	CTGCGCCCA**GAGGTGA**CTCA	-10,315595	ATG	UGL61070.1	hypothetical protein
#21	+	10749	11450	233	ATCTGCA**AAGGAG**AAACACA	-6,610015	ATG	UGL61071.1	single-stranded DNA-binding protein
#22	+	11450	11893	147	TCCGGTCAC**GGTGA**CTTCTG	-5,880535	ATG	UGL61072.1	endonuclease I
#23	+	11895	12335	146	ACCAAAGA**AAGGAGGT**TGAC	-12,108685	ATG	UGL61073.1	lysozyme
#24	+	12411	12953	180	GTTGACA**TAAGGAGGT**TCCT	-13,44061	ATG	UGL61074.1	putative nucleotidyltransferase
#25	+	12940	14631	563	GAATGAAGT**GGAGGTGG**CTG	-12,74128	ATG	UGL61075.1	DNA primase/helicase
#26	+	14645	14848	67	CTAC**TAAGGAG**AACCATCAC	-7,94194	ATG	UGL61076.1	hypothetical protein
#27	+	14914	15375	153	CTCAAT**AGGAG**AACCAAACC	-5,682865	ATG	UGL61077.1	hypothetical protein
#28	+	15386	17530	714	CCAACGAT**AGGAGG**ACGCTG	-8,93096	ATG	UGL61078.1	DNA polymerase
#29	+	17543	17926	127	GTCACTAAT**AGGAGG**CCCTA	-8,93096	ATG	UGL61079.1	hypothetical protein
#30	+	17919	18128	69	GATCGCCC**GAGGTGG**CAGCG	-9,493185	ATG	UGL61080.1	hypothetical protein
#31	+	18138	19088	316	ACGCCATC**TAAGGAGG**GCCT	-11,190035	ATG	UGL61081.1	exonuclease
#32	+	19155	19397	80	TATTCGTTA**AAGGAGG**GCTA	-9,85811	ATG	UGL61082.1	DUF2717 domain-containing protein
#33	+	19400	19684	94	CTGCAA**AGGGAGG**AATGACC	-9,521905	ATG	UGL61083.1	DUF5476 domain-containing protein
#34	+	19681	20124	147	GCCCTGAAGA**AGGT**CACCAA	-3,49838	GTG	UGL61084.1	N-acetyltransferase
#35	+	20096	20401	101	GAAACTG**AAGGAG**AACCCCA	-6,610015	ATG	UGL61085.1	tail assembly protein
#36	+	20415	22040	541	CATTTAATT**GGAGGTGA**CCA	-13,56369	GTG	UGL61086.1	portal protein
#37	+	22110	22985	291	ATTCCCTC**AAGGAG**ACCTCA	-6,610015	ATG	UGL61087.1	capsid assembly protein
#38	+	23088	24131	347	CATGCA**AAGGAG**AACTACAC	-6,610015	ATG	UGL61088.1	major capsid protein
#39	+	24156	24779	207	ACCCAACCCCTT**GAGG**CCCT	-3,60793	TTG	UGL61089.1	tail tubular protein A
#40	+	24789	27209	806	CAAACCGA**TAAGGAGG**GCCT	-11,190035	ATG	UGL61090.1	tail tubular protein B
#41	+	27284	27718	144	CTAC**GGGAGG**GTTTCAACTT	-8,979195	ATG	UGL61091.1	acetyltransferase
#42	+	27729	28310	193	CTGGCTGT**AGGAGGT**ACACC	-11,181535	ATG	UGL61092.1	internal virion protein B
#43	+	28310	30526	738	CCAC**AAGGAGG**CAAGACGTA	-9,85811	ATG	UGL61093.1	internal virion protein C
#44	+	30539	34729	1396	CTCTCTAAT**AGGAGGT**CTAC	-11,181535	ATG	UGL61094.1	internal virion protein D
#45	+	34802	36550	582	TTCAACCAC**AAGGAG**ACTCT	-6,610015	ATG	UGL61095.1	tail fiber protein
#46	+	36587	36946	119	GGCCTCTC**TAAGGAGG**ACTT	-11,190035	ATG	UGL61096.1	hypothetical protein

#47	+	36946	37161	71	CAGATC**AAGG**CGATGAGCTG	-2,174955	ATG	UGL61097.1	holin
#48	+	37158	37415	85	AAGAACA**AAGGAG**ACCCACC	-6,610015	ATG	UGL61098.1	terminase small subunit
#49	+	37418	37873	151	GACGGCGT**GAGG**CACTGACA	-3,60793	GTG	UGL61099.1	i-spanin
#50	+	37569	37823	84	ACGACAACT**GGAGGT**TGAAC	-9,1066	GTG	UGL61100.1	o-spanin
#51	+	37883	39634	583	CGCACAAA**TAAGGAGG**CCCA	-11,190035	ATG	UGL61101.1	terminase large subunit
#52	+	39813	39986	57	CATCA**AAGGAGG**ACAAGACC	-9,85811	ATG	UGL61102.1	hypothetical protein

Supplementary table 2. Predicted ORFs and their functional annotation in the genome of *Pseudomonas* phage Eisa9. Putative Shine-Dalgarno sequences 20 bases upstream of the predicted start codon of each ORF that are complementary to the 16S rRNA tail of *Pseudomonas syringae* pv. tomato (host) are underlined and highlighted in bold. Color coding for ORF product functional groups is as follows: yellow - proteins involved in virion morphogenesis; red— DNA-dependent RNA polymerase; blue —DNA replication, modification and repair; green — lysis; purple — additional functions; gray — hypothetical proteins of unknown function.

ORF	Strand	Start position (bp)	End position (bp)	Product size (aa)	Shine-Dalgarno sequence		Start codon	Protein accession	Predicted function
					20 bp upstream of start codon (G-U wobble base pairs allowed)	DeltaG (kcal/mol; as calculated by free_align.pl -o "20bp" AUUCCUCCACUAG)			
#1	+	21	236	71	AT**AGG**CCAACTGTTAGTAAG	-1,247805	TTG	UGL61103.1	hypothetical protein
#2	+	353	619	88	GCAACCAC**TGA**GTAACGACA	-0,381865	ATG	UGL61104.1	hypothetical protein
#3	+	647	1147	166	AACTGAC**TGAGGG**TTTCATC	-5,05103	ATG	UGL61105.1	hypothetical protein
#4	+	1208	1480	90	TTTGCAT**AGGAG**ACAGGCGC	-5,682865	ATG	UGL61106.1	hypothetical protein
#5	+	1482	1709	75	CGCCAATCGCC**GGGG**CTGAC	-2,833755	ATG	UGL61107.1	hypothetical protein
#6	+	1709	1876	55	ACTAACTAC**GAGGTGGT**GTA	-10,859585	ATG	UGL61108.1	hypothetical protein
#7	+	1869	2192	107	CCAACTGCC**GGGTG**CTGACT	-5,64358	ATG	UGL61109.1	hypothetical protein
#8	+	2449	2946	165	GCAACAACATC**AGGTGA**ACT	-7,95547	ATG	UGL61110.1	putative acetyltransferase
#9	+	2937	3359	140	GTATTCAT**GGAG**CTGCCAGC	-3,60793	ATG	UGL61111.1	hypothetical protein
#10	+	3343	3537	64	GATAGCGGC**GGA**CCAAGAAC	-1,532995	ATG	UGL61112.1	hypothetical protein
#11	+	3534	3794	86	ACCTTC**AAGGAG**TACAGGCC	-6,610015	ATG	UGL61113.1	hypothetical protein
#12	+	3791	4669	292	CTGATGAT**GGAG**TGTGACGA	-3,60793	ATG	UGL61114.1	hypothetical protein
#13	+	4669	4977	102	CGTCAAGT**GGAG**TTCGTGTA	-3,60793	ATG	UGL61115.1	hypothetical protein
#14	+	4982	5242	86	CACTCT**GGAGG**GCTAAGCCC	-6,856025	ATG	UGL61116.1	hypothetical protein
#15	+	5325	5807	160	CCAAACGA**AAGGA**TTACACC	-4,53508	ATG	UGL61117.1	hypothetical protein
#16	+	5807	6514	235	GCCC**TGATC**GGCAGCCTGTA	-3,840985	ATG	UGL61118.1	hypothetical protein
#17	+	6511	7314	267	CTGGTGTTCT**GGAG**TAAGCA	-3,60793	TTG	UGL61119.1	putative DNA primase
#18	+	7480	7668	62	TCCGCAC**AGGAG**ACGCGAGC	-5,682865	ATG	UGL61120.1	hypothetical protein
#19	+	7658	8944	428	TAAACTGGT**GGAGGT**ACACG	-9,1066	ATG	UGL61121.1	DNA helicase
#20	+	8931	9161	76	GGATGCAAC**GGGAGG**ACGTT	-8,979195	ATG	UGL61122.1	hypothetical protein
#21	+	9158	11494	778	CTGTGCAAGGCGT**GGTG**CGC	-3,52041	ATG	UGL61123.1	DNA polymerase
#22	+	11503	12390	295	CAATACGA**GTGA**GAGAAGAC	-2,63244	ATG	UGL61124.1	hypothetical protein

#23	+	12390	13400	336	GCGCTGGCC**GGT**ATCCTCTG	-1,423445	ATG	UGL61125.1	exonuclease
#24	+	13402	13743	113	TCGCACCGATCA**GGGT**TAAA	-3,546615	ATG	UGL61126.1	putative recombination endonuclease
#25	+	13743	14717	324	AAAGCAA**AGGT**ATCGAAATA	-3,49838	ATG	UGL61127.1	hypothetical protein
#26	+	14717	15274	185	CGCTGGGTC**GGGAG**CCTCTA	-5,7311	ATG	UGL61128.1	DUF5664 domain-containing protein
#27	+	15261	15446	61	TCTCCTGT**TGAGGA**TCAAAC	-5,87344	ATG	UGL61129.1	hypothetical protein
#28	+	15446	16348	300	ATCTCT**GGAGGTGA**AATCTG	-13,56369	ATG	UGL61130.1	DNA ligase
#29	+	16349	18772	807	AGACCGAGCCGA**GTGA**GTAA	-2,63244	ATG	UGL61131.1	DNA-dependent RNA polymerase
#30	+	18830	19132	100	ACTTGCC**GGAGG**ATCGCTTC	-6,856025	ATG	UGL61132.1	hypothetical protein
#31	+	19119	19601	160	GCGGACAGT**GGAGG**CCGAAC	-6,856025	TTG	UGL61133.1	hypothetical protein
#32	+	19603	20079	158	CGTCAAAC**GGAG**TATCTGAT	-3,60793	ATG	UGL61134.1	hypothetical protein
#33	+	20076	21590	504	AGCG**GATC**GAGCGTCCGTCT	-1,74402	GTG	UGL61135.1	portal protein
#34	+	21587	22372	261	GTACCC**GGAG**ATACTGCACA	-3,60793	ATG	UGL61136.1	scaffolding protein
#35	+	22449	23456	335	CACCACA**TAAGGA**TTCCACA	-5,867005	ATG	UGL61137.1	major capsid protein
#36	+	23531	24133	200	TTGCGTTT**AGGAG**ATAGCCT	-5,682865	ATG	UGL61138.1	tail tubular protein A
#37	+	24130	26661	843	CCGAC**AGGA**CGGCGATTCAG	-3,60793	ATG	UGL61139.1	tail tubular protein B
#38	+	26665	27414	249	TCACTC**GGAGG**ACTTAACAC	-6,856025	ATG	UGL61140.1	internal virion protein
#39	+	27423	29675	750	AAACTTCTGT**AGGAGGT**AAC	-11,181535	ATG	UGL61141.1	internal virion protein
#40	+	29684	33511	1275	CCTAATAAG**TGAGGG**CCACA	-5,05103	ATG	UGL61142.1	internal virion protein
#41	+	33572	35464	630	AACCAATTCT**GGAG**AACCAA	-3,60793	ATG	UGL61143.1	tail fiber protein
#42	+	35474	35818	114	GAGTTTCG**TAAGGAG**ATACA	-7,94194	ATG	UGL61144.1	putative tail fiber assembly protein
#43	+	35815	35997	60	CAAATCA**AGGTGAT**GCAAGA	-9,054465	ATG	UGL61145.1	holin
#44	+	35984	36286	100	CTACGAGA**AGGT**CAAGAGCA	-3,49838	ATG	UGL61146.1	terminase small subunit
#45	+	36286	38064	592	GCCCAAAT**GGGT**ATTCACTG	-3,546615	ATG	UGL61147.1	terminase large subunit
#46	+	38068	38328	86	CTCGTAA**AAGGA**AATGATCC	-4,53508	ATG	UGL61148.1	hypothetical protein
#47	+	38340	38639	99	GACCTG**TAAGGAG**CAACACC	-7,94194	ATG	UGL61149.1	hypothetical protein
#48	+	38643	39143	166	ACGCT**GTG**CTGTCCTAAGTC	-0,272315	ATG	UGL61150.1	endolysin
#49	+	39140	39445	101	CTCTGG**AAGGAGG**GTTATCA	-9,85811	ATG	UGL61151.1	putative i-spanin
#50	+	39354	39584	76	CACCCGC**AAGGAGGTGA**GCG	-16,565775	ATG	UGL61152.1	putative o-spanin
#51	+	39675	40163	162	AGGTCACCT**AGGA**CAAAAAA	-3,60793	CTG	UGL61153.1	hypothetical protein

Supplemetary Figure S1. Viridic heatmap showing pairwise intergenomic distances (%) of *Pseudomonas* phages Eir4 and Eisa9 with other related phages. Labels of *Pseudomonas* infecting *Autographivirdae* phages with a standing in the official phage taxonomy (subset from the ICTV virus metadata resource; n=49) are in black font color. Labels of ten phages most similar to Eir4 that were not found as a part of *Pseudomonas*-infecting *Autographiviridae* phage VMR subset are in red font color (n=10), label of Eir4 is in red and is also made bold. Labels of ten phages most similar to Eisa9 that were not found as a part of *Pseudomonas*-infecting *Autographiviridae* phage VMR subset are in blue font color (n=10), label of Eisa9 is also made bold. Red rectangle indicates a cluster of phage genomes that are either officially recognized as representatives of genus *Ghunavirus* or can be proposed for inclusion in it. Maroon rectangle indicates *Pseudomonas* phage isolate genomes that belong to the yet unproposed same phage species as Eir4. Purple rectangle indicates *Pseudomonas* phages PollyC and Eisa9, which can probably be proposed as an exemplar isolate for the creation of a novel species in the *Polyceevirus* genus.

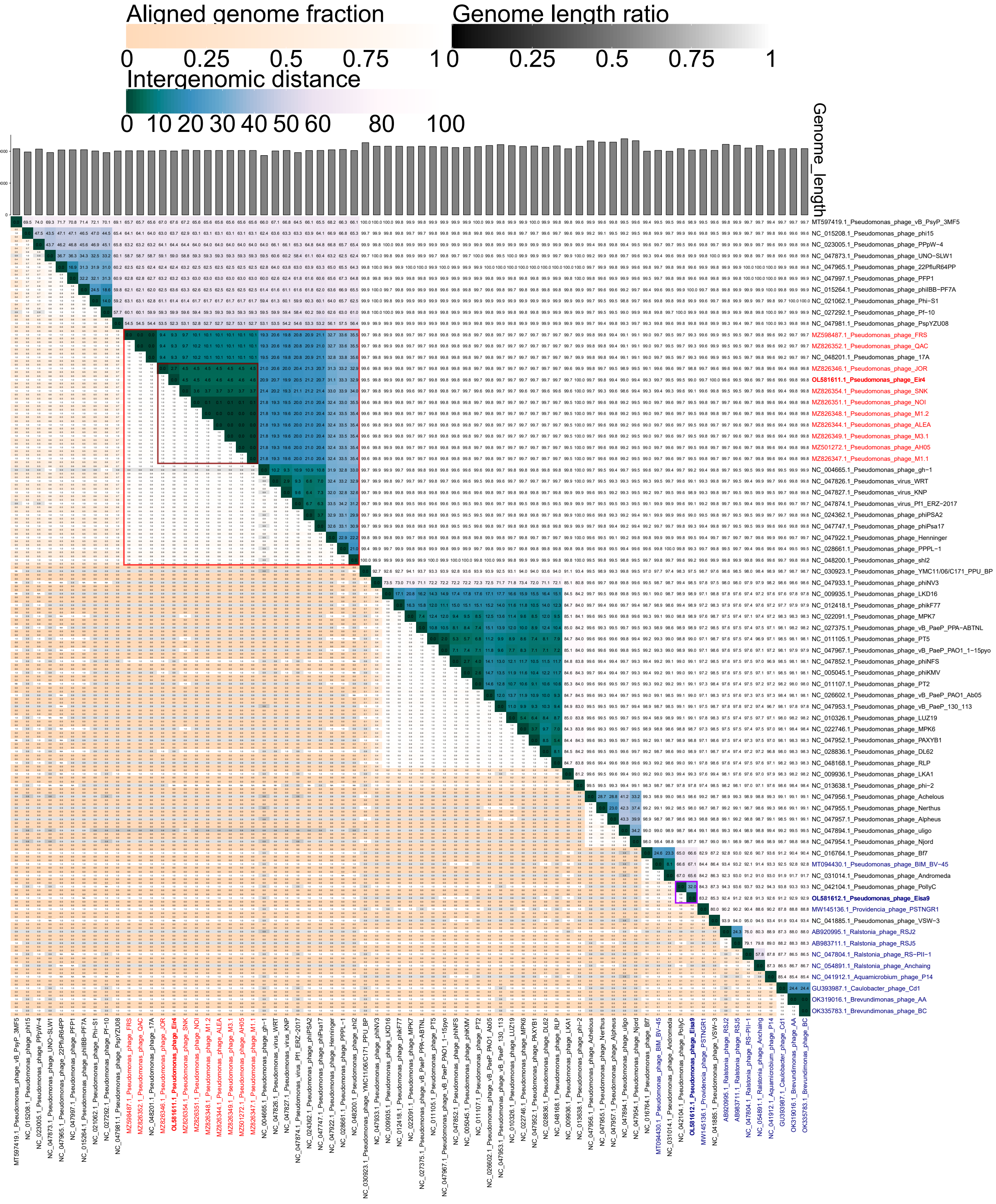
Aligned genome fraction
0 0.25 0.5 0.75 1
Genome length ratio
0 0.25 0.5 0.75 1
Intergenomic distance
0 10 20 30 40 50 60 80 100
Genome_length
40000
20000
0
MT597419.1_Pseudomonas_phage_vB_PsyP_3MF5
NC_015208.1_Pseudomonas_phage_phi15
NC_023005.1_Pseudomonas_phage_PPpW-4
NC_047873.1_Pseudomonas_phage_UNO-SLW1
NC_047965.1_Pseudomonas_phage_22PfluR64PP
NC_047997.1_Pseudomonas_phage_PFP1
NC_015264.1_Pseudomonas_phage_philBB-PF7A
NC_021062.1_Pseudomonas_phage_Phi-S1
NC_027292.1_Pseudomonas_phage_Pf-10
NC_047981.1_Pseudomonas_phage_PspYZU08
MZ598487.1_Pseudomonas_phage_FRS
MZ826352.1_Pseudomonas_phage_QAC
NC_048201.1_Pseudomonas_phage_17A
MZ826346.1_Pseudomonas_phage_JOR
OL581611.1_Pseudomonas_phage_Eir4
MZ826354.1_Pseudomonas_phage_SNK
MZ826351.1_Pseudomonas_phage_NOI
MZ826348.1_Pseudomonas_phage_M1.2
MZ826344.1_Pseudomonas_phage_ALEA
MZ826349.1_Pseudomonas_phage_M3.1
MZ501272.1_Pseudomonas_phage_AH05
MZ826347.1_Pseudomonas_phage_M1.1
NC_004665.1_Pseudomonas_phage_gh-1
NC_047826.1_Pseudomonas_virus_WRT
NC_047827.1_Pseudomonas_virus_KNP
NC_047874.1_Pseudomonas_virus_Pf1_ERZ-2017
NC_024362.1_Pseudomonas_phage_phiPSA2
NC_047747.1_Pseudomonas_phage_phiPsa17
NC_047922.1_Pseudomonas_phage_Henninger
NC_028661.1_Pseudomonas_phage_PPPL-1
NC_048200.1_Pseudomonas_phage_shl2
NC_030923.1_Pseudomonas_phage_YMC11/06/C171_PPU_BP
NC_047933.1_Pseudomonas_phage_phiNV3
NC_009935.1_Pseudomonas_phage_LKD16
NC_012418.1_Pseudomonas_phage_phikF77
NC_022091.1_Pseudomonas_phage_MPK7
NC_027375.1_Pseudomonas_phage_vB_PaeP_PPA-ABTNL
NC_011105.1_Pseudomonas_phage_PT5
NC_047967.1_Pseudomonas_phage_vB_PaeP_PAO1_1-15pyo
NC_047852.1_Pseudomonas_phage_phiNFS
NC_005045.1_Pseudomonas_phage_phiKMV
NC_011107.1_Pseudomonas_phage_PT2
NC_026602.1_Pseudomonas_phage_vB_PaeP_PAO1_Ab05
NC_047953.1_Pseudomonas_phage_vB_PaeP_130_113
NC_010326.1_Pseudomonas_phage_LUZ19
NC_022746.1_Pseudomonas_phage_MPK6
NC_047952.1_Pseudomonas_phage_PAXYB1
NC_028836.1_Pseudomonas_phage_DL62
NC_048168.1_Pseudomonas_phage_RLP
NC_009936.1_Pseudomonas_phage_LKA1
NC_013638.1_Pseudomonas_phage_phi-2
NC_047956.1_Pseudomonas_phage_Achelous
NC_047955.1_Pseudomonas_phage_Nerthus
NC_047957.1_Pseudomonas_phage_Alpheus
NC_047894.1_Pseudomonas_phage_uligo
NC_047954.1_Pseudomonas_phage_Njord
NC_016764.1_Pseudomonas_phage_Bf7
MT094430.1_Pseudomonas_phage_BIM_BV-45
NC_031014.1_Pseudomonas_phage_Andromeda
NC_042104.1_Pseudomonas_phage_PollyC
OL581612.1_Pseudomonas_phage_Eisa9
MW145136.1_Providencia_phage_PSTNGR1
NC_041885.1_Pseudomonas_phage_VSW-3
AB920995.1_Ralstonia_phage_RSJ2
AB983711.1_Ralstonia_phage_RSJ5
NC_047804.1_Ralstonia_phage_RS-PII-1
NC_054891.1_Ralstonia_phage_Anchaing
NC_041912.1_Aquamicrobium_phage_P14
GU393987.1_Caulobacter_phage_Cd1
OK319016.1_Brevundimonas_phage_AA
OK335783.1_Brevundimonas_phage_BC

Supplementary Figure S2. Terminase large subunit protein amino acid sequence maximum-likelihood tree for prediction of packaging strategy employed by phages Eir4 and Eisa9. The tree is drawn to scale, branch lengths correspond to the number of amino acid differences per site. Tips are labeled as “protein accession | originating phage | packaging strategy/genome termini type employed by the phage”, labels of the tips representing the amino acid sequences of Eir4 and Eisa9 proteins are coloured in red and blue, respectively. The tree is midpoint rooted and the distal nodes of branches with >=95% UFBoot support (out of 1000 replicates) are indicated by green squares. Maroon rectangle indicates the clade containing MRCA of T7 type SDTR packaging strategy employing phages and its children. “Unknown*” indicates that the TerL sequence comes from a phage for which the packaging strategy is unknown. LDTR stands for long direct terminal repeats, SDTR - for short direct terminal repeats.

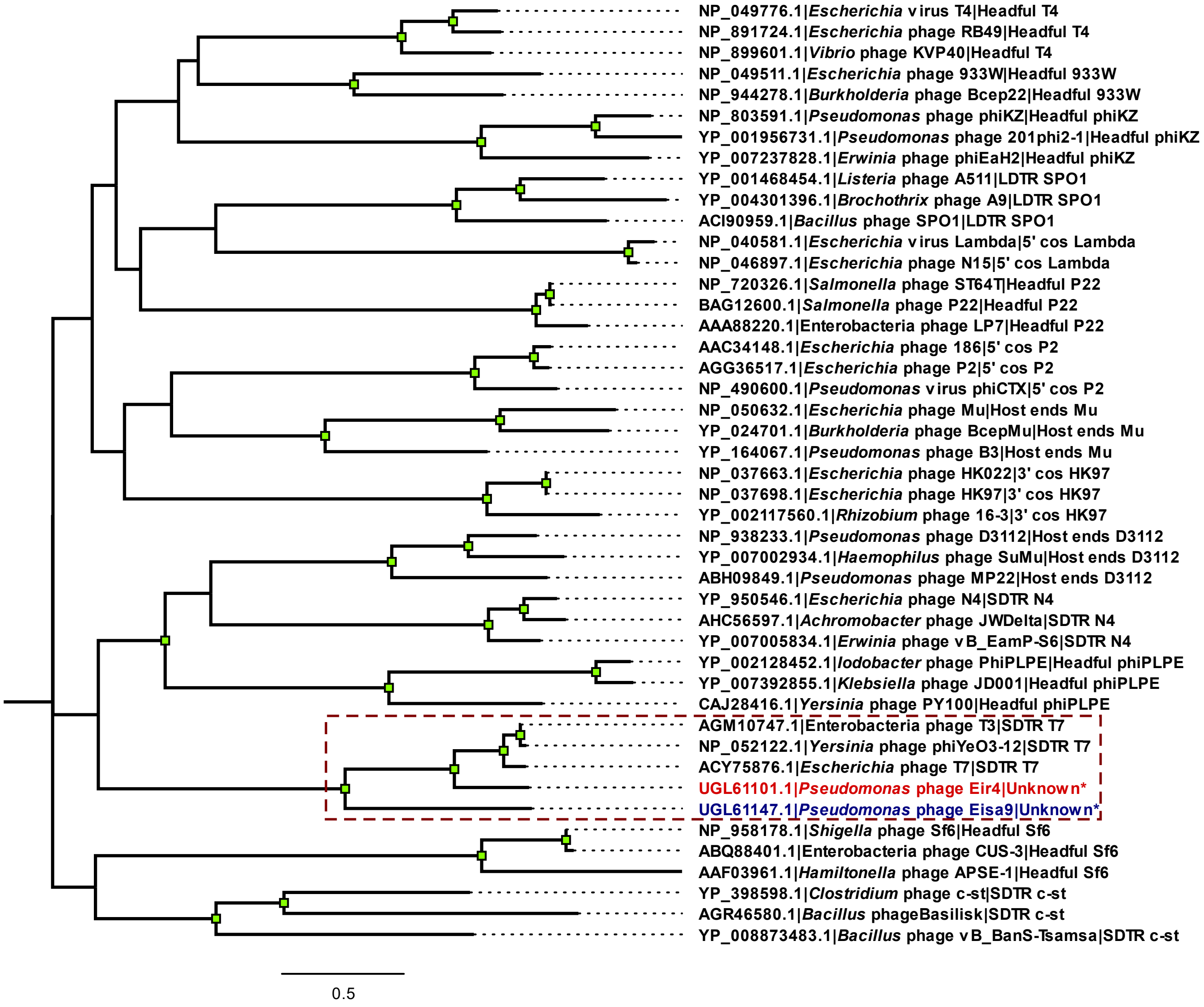

Supplementary Figure S3. Pairwise genome nucleotide sequence comparison of *Autographivirdae* phage T7 as an exemplar *Autographivirdae* phage, phage 17A (representative of *Ghunavirus* genus) as the closest Eir4 relative with the standing in the current phage taxonomy, *Pseudomonas* phage Eir4 itself and eight phages that belong to the same putative novel species in genus *Ghunavirus* as Eir4 using BLASTN. Genome representations are drawn to scale with the scale bar indicating 2000 base pairs. Arrows represent open reading frames and point in the direction of transcription. Color-coding is based on the functional groups of the respective ORF putative products according to the legend and ORFs of Eir4 with functional prediction are annotated by the order of their appearance in the genome (Supplementary tables 1 and 2). Gray boxes represent regions of similarity between the genomes and are coloured in gradient with darker shades of gray representing higher region identity. The figure was generated using EasyFig (Sullivan et al., 2011).

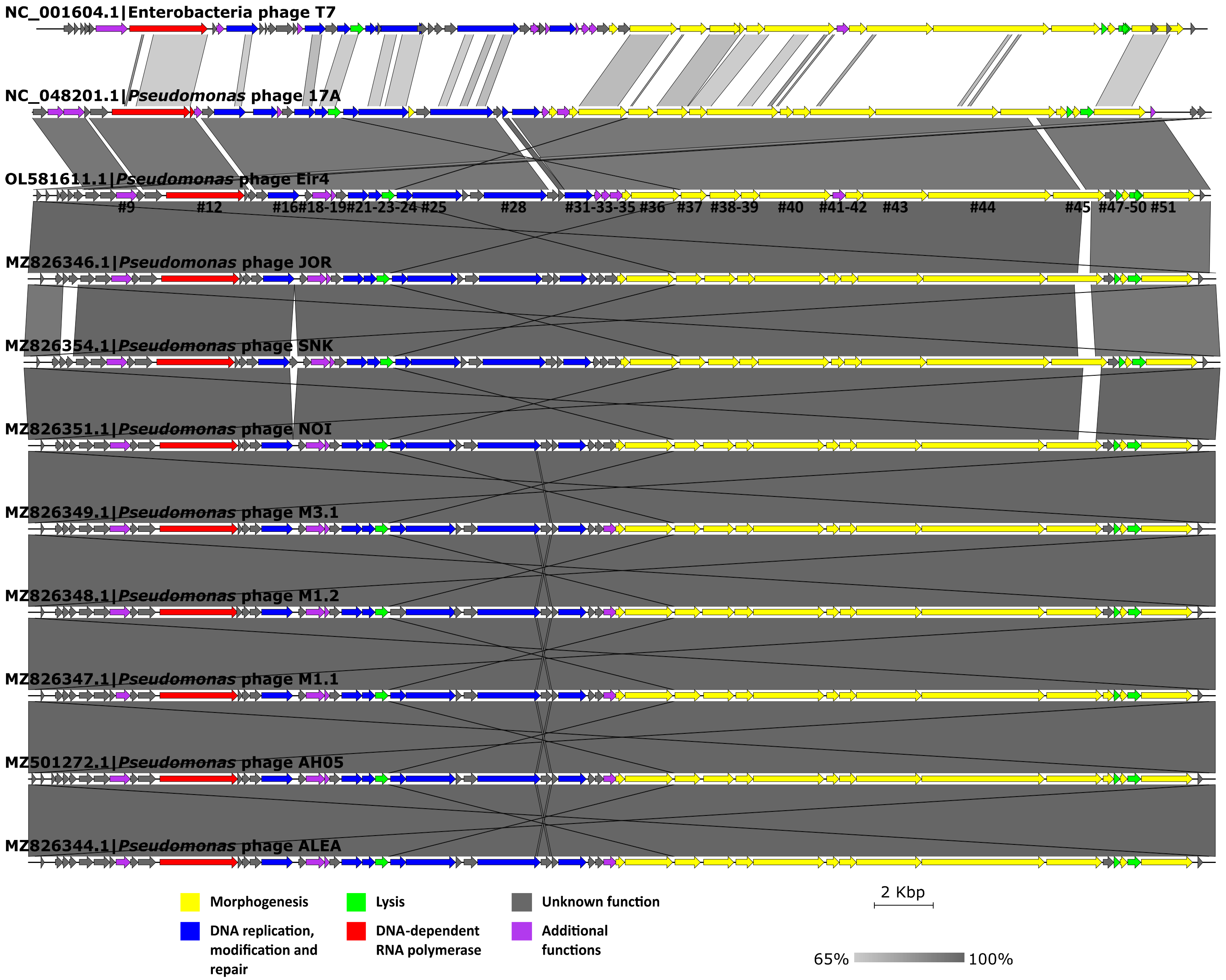

NC_001604.1|Enterobacteria phage T7
NC_048201.1|Pseudomonas phage 17A
OL581611.1|Pseudomonas phage Eir4
#9
#12
#16#18-19#21-23-24 #25
#28
#31-33-35 #36
#37 #38-39
#40
#41-42
#43
#44
#45 #47-50 #51
MZ826346.1|Pseudomonas phage JOR
MZ826354.1|Pseudomonas phage SNK
MZ826351.1|Pseudomonas phage NOI
MZ826349.1|Pseudomonas phage M3.1
MZ826348.1|Pseudomonas phage M1.2
MZ826347.1|Pseudomonas phage M1.1
MZ501272.1|Pseudomonas phage AH05
MZ826344.1|Pseudomonas phage ALEA
Morphogenesis
Lysis
Unknown function
DNA replication, modification and repair
DNA-dependent RNA polymerase
Additional functions
2 Kbp
65%
100%
